# Altered FGF expression profile in human scalp-derived fibroblasts upon WNT activation: implication of their role to provide folliculogenetic microenvironment

**DOI:** 10.1186/s41232-020-00141-8

**Published:** 2020-09-21

**Authors:** Misaki Kinoshita-Ise, Aki Tsukashima, Tomonari Kinoshita, Yoshimi Yamazaki, Manabu Ohyama

**Affiliations:** 1 Department of Dermatology, Kyorin University School of Medicine, 6-20-2 Shinkawa, Mitaka-shi, Tokyo, 181-8611 Japan; 2 Department of Dermatology, Keio University School of Medicine, 35 Shinanomachi, Shinjyuku, Tokyo, 160-8582 Japan; 3 Division of Cellular Signaling Institute for Advanced Medical Research, Keio University School of Medicine, 35 Shinanomachi, Shinjuku-ku, Tokyo, 160-8582 Japan

**Keywords:** Hair regeneration, Fibroblast growth factors, Dermal papillae cells, Dermal sheath cells, Keratinocytes, Fibroblasts, WNT signaling, FGF7, FGF9

## Abstract

**Background:**

Hair follicle (HF) formation and growth are sustained by epithelial-mesenchymal interaction via growth factors and cytokines. Pivotal roles of FGFs on HF regeneration and neogenesis have been reported mainly in rodent models. FGF expression is regulated by upstream pathways, represented by canonical WNT signaling; however, how FGFs influence on human folliculogenesis remains elusive. The aim of this study is to assess if human scalp-derived fibroblasts (sFBs) are able to modulate their FGF expression profile in response to WNT activation and to evaluate the influence of WNT-activated or suppressed FGFs on folliculogenesis.

**Methods:**

Dermal papilla cells (DPCs), dermal sheath cells (DSCs), and sFBs were isolated from the human scalp and cultured independently. The gene expression profile of FGFs in DPCs, DSCs, and sFBs and the influence of WNT activator, CHIR99021, on FGF expression pattern in sFBs were evaluated by reverse transcription polymerase chain reaction, which were confirmed at protein level by western blotting analysis. The changes in the expression of DPC or keratinocyte (KC) biomarkers under the presence of FGF7 or 9 were examined in both single and co-culture assay of DPCs and/or KCs. The influence of FGF 7 and FGF 9 on hair morphogenesis and growth was analyzed in vivo using mouse chamber assay.

**Results:**

In single culture, sFBs were distinguished from DPCs and DSCs by relatively high expression of *FGF5* and *FGF18*, potential inducers of hair cycle retardation or catagen phase. In WNT-activated state, sFBs downregulated *FGF7* while upregulating *FGF9*, a positive regulator of HF morphogenesis, FGF16 and *FGF20* belonging to the same FGF subfamily. In addition, CHIR99021, a WNT activator, dose-dependently modulated FGF7 and 9 expression to be folliculogenic. Altered expressions of FGF7 and FGF9 by CHIR99021 were confirmed at protein level. Supplementation of FGF9 to cultured DPCs resulted in upregulation of representative DP biomarkers and this tendency was sustained, when DPCs were co-cultured with KCs. In mouse chamber assay, FGF9 increased both the number and the diameter of newly formed HFs, while FGF7 decreased HF diameter.

**Conclusion:**

The results implied that sFBs support HF formation by modulating regional FGF expression profile responding to WNT activation.

## 1. Background

The ultimate goal of regenerative medicine is to reconstruct fully functional bioengineered organs which can substitute missing or disabled ones after disease, injury, or aging [1]. Longtime focuses of regenerative medicine had been mainly on the preparation of the cells or their assembly to reconstitute target organs [2], and little attention has been paid to the role of microenvironment enabling cell-cell interactions [3]. In the case of hair follicle (HF) regeneration, the main subjects have been the preparation of both epithelial and mesenchymal (dermal) HF stem or progenitor cells and the methodologies to elicit folliculogenic epithelial-mesenchymal interactions [4]. The main body of HF comprises keratinocytes (KCs), melanocytes, and dermal cells, namely, dermal papilla cells (DPCs) and dermal sheath cells (DSCs) possessing hair inductive capacities. The stem/progenitor cell populations for each cell subset has been identified [4] and predominantly investigated in the light of efficient isolation, maintenance of intrinsic properties during in vitro expansion, and development of methodology to maximize their interactions [4]. Although HF is surrounded by the dermis which is rich in another mesenchymal component of dermal fibroblasts, the role of these cells has not been highly regarded in HF regenerative medicine. A recent study suggested that fibroblast growth factor 9 (FGF9) secreted from perifollicular dermal fibroblasts in response to upstream WNT activation plays a key role in the wound-induced model of HF neogenesis in mouse [5]. In contrast, FGF7 has been shown to block HF induction and promote interfollicular epidermal fate in mouse skin [6]. Presumably, via FGFs, dermal fibroblasts actively engage in the regulation of HF formation. Mammalian FGF is comprised of 23 factors and can be subdivided into 7 subfamilies based on its structural similarity, biochemical functions, and evolutionary relationships [7]. Considering their diversity, it is reasonable to speculate that identical molecules may exhibit respective roles in other species. Yet, past studies mainly adopted murine knockout models, allowing the functional dissection of limited number of FGFs. Whether human dermal fibroblasts, especially those residing in the scalp dermis, produce FGFs, let alone their FGF production profiles, is still ill-investigated. Therefore, we attempted to investigate, firstly, if human scalp-derived dermal fibroblasts (sFBs) are able to express FGFs similarly to DPCs or DSCs which are already known to produce them and, if yes, whether they modulate their FGF expression profile upon WNT activation. Functionality of identified FGFs was also assessed using the chamber hair reconstitution assay.

## 2. Methods

### 2.1. Preparation of cells from the human scalp

Human HFs and surrounding dermal tissue were collected from the pieces of intact scalp skin obtained during the surgical removal of benign skin tumors. The dermal papilla and the dermal sheath were micro-dissected from HFs and sFBs were isolated from the dermis around HFs, following previously described methods [8, 9].

### 2.2. Cell culture

DPCs DSCs, and sFBs were obtained from the outgrowth from micro-dissected tissue placed in Dulbecco’s modified Eagle’s medium (DMEM; Invitrogen, CA, USA) with 10% fetal bovine serum (FBS), penicillin, and streptomycin and subsequently cultured. Purchased KCs (CellnTEC, Bern, Switzerland) were cultured in KCs’ serum-free medium (Invitrogen, CA, USA). The cultured cells were incubated at 37 °C in air containing 5% CO_2_. Culture medium was changed every 3 or 4 days, and cells were passaged at 70–80% confluency. The cells at passage 2 or 3 were exclusively used in this study.

### 2.3. WNT activation in vitro

sFBs were cultured as described above until 70% confluency and then the medium was changed to DMEM containing 1, 5, or 10 μM CHIR99021 (Cayman Chemical, MI, USA) solved in dimethyl sulfoxide (DMSO) and cultured for 7 days to activate WNT signaling.

### 2.4. Co-culture of KCs and DPCs

The established co-culture method was utilized [10]. Briefly, KCs and DPCs were cultured in a method described above and harvested. Then, DPCs were seeded onto upper insert wells (Falcon; CORNING, NY, USA) and KCs onto lower wells with collagen type I coating (IWAKI, Tokyo, Japan). Co-culture was carried out for 48 h in DMEM supplemented with human recombinant FGF7 (50 ng/mL), FGF 9 (50 ng/mL), or Phosphate-buffered saline (PBS) (control) before total RNA was extracted from each cell subset using RNeasy Protect Mini Kit (QIAGEN, Hilden, Germany) following the manufacturer’s protocol.

### 2.5. Reverse transcription-polymerase chain reaction

**Fig. 1:**
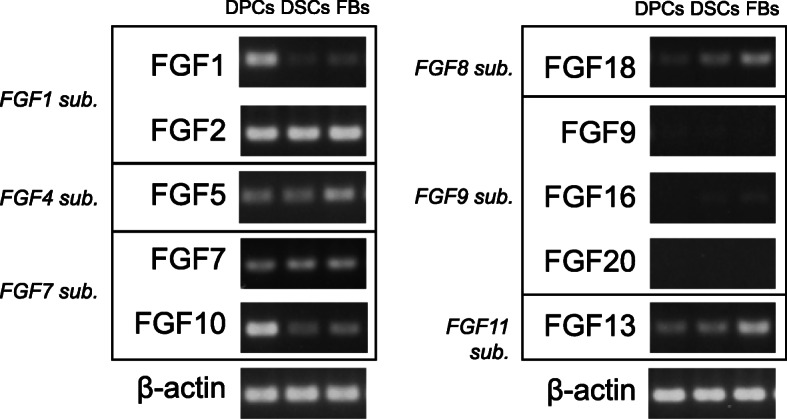
FGF gene expression profiles in human scalp-derived dermal cell subsets. *FGF1* and *FGF10* were intensely expressed in DPCs compared to DSCs and sFBs. *FGF5*, *FGF13*, and *FGF18* showed high expression in sFBs. *FGF9 *as well as *FGF16* and *FGF20,* belonging to the same *FGF9* subfamilywere barely expressed in sFBs. Samples were obtained from a single donor

Total RNA was extracted from each cell lineage and cDNA was synthesized using the Superscript III First Strand Synthesis SuperMix (Invitrogen, CA, USA) according to the manufacturer’s protocol. Semi-quantitative reverse transcription-polymerase chain reaction (RT-PCR) was performed using HotStarTaq Master Mix Kit (QIAGEN, Hilden, German) with the cycle condition consisted of an initial activation of 15 min at 95 °C, then 34 cycles of denaturing for 15 s at 94 °C and annealing for 30 s at 55 °C and extension for 60 s at 72 °C. One dataset was obtained using DPCs, DSCs, and FBs from an identical donor (Fig. 1). Quantitative RT-PCR (qRT-PCR) were performed as previously described [11, 12], using SYBER select Master mix (Thermo Fisher Scientific, MA, USA) on the Applied Biosystems StepOnePlus Real-Time PCR system (Thermo Fisher Scientific, MA, USA). Cycling conditions consisted of an initial activation of 10 min at 95 °C, then 40 cycles of denaturing for 15 s at 95 °C and annealing and extension for 60 s at 60 °C. Three experiments were performed with sFBs from different patients to examine the alteration of each FGFs expression and two experiments to examine the alteration of FGF7 and FGF9 in sFBs under supplementation of different doses of CHIR 99021. Four experiments with DPs and KCs from different patients were conducted for singly cultured DPCs, co-cultured DPs, and co-cultured KCs, and three experiments were performed for singly cultured KCs. The primers used in RT-PCR and quantitative RT-PCR are listed in Supplementary Table S1 and S2 respectively.

### 2.6. Western blot

Lysates from sFBs were prepared using RIPA buffer (25 mM Tris–HCl [pH 7.6], 150 mM sodium chloride, 1% NP-40, 1% sodium deoxycholate, and 0.1% sodium dodecyl sulfate (SDS)) containing protease inhibitors (Roche, Basel, Switzerland) according to the standard method [13], followed by centrifugation at 14,000×*g* for 15 min at 4 °C, and the concentration of each sample using a Bio-Rad protein assay kit (Bio-Rad, Tokyo, Japan) with bovine serum albumin as a standard. The sample was electrophoresed in 10% SDS-polyacrylamide gel and transferred to a nitrocellulose membrane and incubated with primary antibodies: FGF7 (1:1000; abcam, Tokyo, Japan), FGF9 (1:1000; abcam, Tokyo, Japan), and GAPDH (1:5000; Sigma-Aldrich, Tokyo, Japan). After washed by TBST (20 mM Tris–HCl, 150 mM NaCl, and 0.02% Tween-20, pH 7.4), the blots were incubated with secondary antibodies conjugated with horseradish peroxidase (1:4000, anti-rabbit; GE Healthcare, Tokyo, Japan) for 1 h at room temperature. The bands were detected using ECL-Plus Substrate (GE Healthcare, IL, USA) and exposed to Hyperfilm (GE Healthcare, IL, USA). The data was obtained from an experiment using samples from a single donor.

### 2.7. In vivo hair induction assay

Following the previously described protocol for the chamber assay [4], epidermal and dermal cells were isolated from BALB/cCrSlc mouse embryos (E18–19) and transplanted into the chambers grafted onto the fascia of SCID mice. FGF7 (5 μg/mL), FGF9 (5 μg/mL), or PBS as control was injected at the dose of 100 μL through minute pole on the top of silicon chambers [14] every other day for 2 weeks, before the silicon chambers were removed. Four weeks after the removal, the regenerated back skin was harvested for the histopathological analysis. The experiments were repeated three times (*n* = 7 for each group).

### 2.8. Histopathological analysis

Specimens were fixed by 20% acid-alcohol-formalin for at least 24 h and embedded in paraffin and sectioned. Each section was stained with hematoxylin and eosin solution for histopathological analysis.

### 2.9. Statistical analysis

Numerical results are presented as mean with standard error. Statistical analysis was performed using SPSS Statistics 23.0 software (IBM Corp, NY, USA). The comparison between two groups or among three groups were respectively conducted with two-sided Student’s *t* test or one-way ANOVA. A *p* value less than 0.05 was considered to be significant.

## 3. Results

### 3.1. Human scalp-derived dermal cell subsets demonstrated distinct FGF expression profiles

Our review of the literature elucidated that nearly 10 FGFs have been reported in association with HF biology (Table 1); however, most studies focused on FGFs in KCs or DPCs [6, 15, 17–38]. As a preliminary investigation to probe uniqueness of FGF expression profiles of three human scalp-derived dermal cell populations, total RNA was respectively extracted from DSCs, DPCs, and sFBs of a single donor to conduct semi-quantitative RT-PCR analysis. Intriguingly, each cell subset exhibits distinctive FGF expression profile. Human FGFs are largely divided into 7 subgroups based on their biochemical characteristics, namely, *FGF1* (1, 2), *FGF4* (4, 5, 6), *FGF7* (3, 7, 10, 12), *FGF8* (8, 7, 18), *FGF9* (9, 16, 20), *FGF11* (11, 12, 13, 14), and *FGF15/19* (19, 21, 23) subfamilies [39]. The representative results of RT-PCR analyses are presented in Fig. 1. Each human scalp-derived dermal cell subpopulations demonstrated a unique FGF gene expression profile. Notably, the FGF gene expression pattern of sFBs was clearly distinct from those in DPCs and DSCs, with relatively high expression of *FGF5*, *18* and *13* (Fig. 1).* FGF9*, *16*, and *20* are belonging to the *FGF 9* subfamily [39], which were just weakly expressed in three dermal cell lineages. Among them, *FGF16* expression seems to be characteristic in sFBs compared to DPCs and DSCs. *FGF7* seemed to be expressed  ubiquitously in three lineages. *FGF2*, which is crucial in tissue repair [40], was highly expressed in all three dermal cell subgroups. *FGF1* and *10* was intensely expressed in DPCs compared to other HF-associated dermal cells.

**Table 1:** Summary of the role of fibroblast growth factors (FGFs) which influence on regeneration/neogenesis and growth/maintenance of hair follicle (HF).

Subfamily	Role on HF		Detailed function	Animals	Location	Ref.
	Regeneration Neogenesis	Growth Maintenance				
**FGF1s (1, 2)**						
FGF1	+	+	Relates with HF differentiation, prevents radiation induced apoptosis of HFs	Ovine, mouse	KC	[15, 16]
FGF2	+	+	Proliferates HF cells, induce HFs, lengthens anagen	Ovine, mouse	KC, DP	[15, 17–19]
**FGF4s (4, 6, 5)**						
FGF5		-	Induces catagen, inhibits hair growth by blocking DPC activation	Mouse, rat, cetasean, human		[20–23]
**FGF7s (3, 7, 10, 22)**						
FGF7	-	+	Protects HF from damage, lengthens anagen, blocks hair follicle induction, related HF texture, proliferates HGs, regulates HF structure	Human, mouse	DP, ORS	[6, 19, 24–28]
FGF10		+	Regulates HF structure, required for HF formation/morphogenesis	Mouse	DP	[26, 29, 30]
**FGF9s (9, 16, 20)**						
FGF9	+		Induces HF neogenesis after wounding	Mouse	γδT cells	[5, 31]
FGF20	+		Governs dermal condensation feather placode induction	Mouse, chicken	Hair placode	[32, 33]
**FGF8s (8, 17, 18)**						
FGF18		−	Induces telogen, stem cell quiescence	Mouse	KC	[34–36]
**FGF19s (19, 21, 23)**						
**FGF11s (11, 12, 13, 14)**						
FGF13		+	Regulates function of bulge reduced in hypertrichosis	Mouse	Bulge region	[37]

### 3.2. Influence of WNT activation on FGF expression in sFBs

**Fig. 2:**
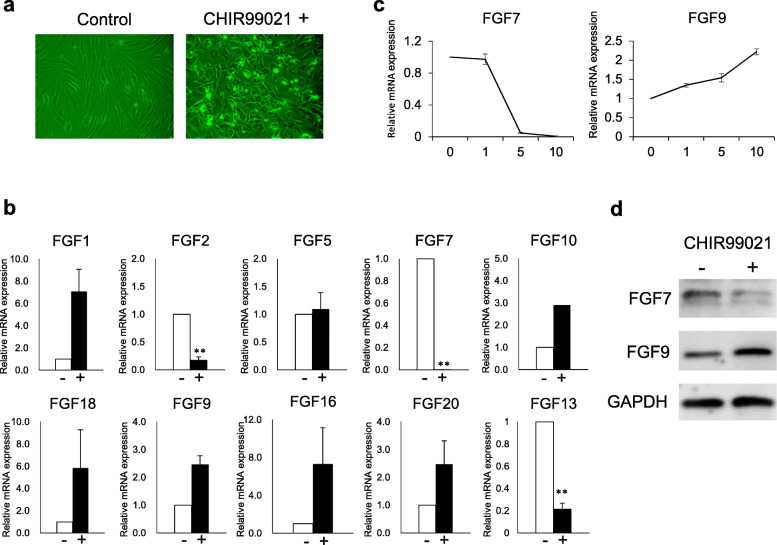
Effect of WNT activation on morphology and gene expression of FGFs in human-scalp derived-fibroblasts. **a** Morphological alteration of human scalp-derived fibroblasts (sFBs) after 1 week cultivation with a WNT agonist. **b** Influence of WNT activation on gene expression of representative FGFs in sFBs. *p < 0.05. **p < 0.01. The data were obtained by three respective experiments. **c** Dose-dependent changes in *FGF7/9* expression after 1 week culture with different concentration of CHIR99021. The data were obtained by two respective experiments. **d** Western blotting analyses of FGF7/9 expression in sFBs after 1 week culture with CHIR99021

In HF morphogenesis, intense WNT activation is pivotal [41]. As elucidation of potential role of sFBs in HF formation is central in this study, the effect of CHIR99021, an established WNT signaling activator [42], on sFBs were assessed. On WNT activation, sFBs greatly changed their morphology, suggesting major alteration in their biological properties (Fig. 2a). FGF gene expression analysis demonstrated that some FGFs are differentially expressed before and after WNT activation. *FGF1*, *10*, *18*, *9*, *16*, and *20* were tendentiously upregulated by the folds of 7.07 ± 2.00, 2.90 ± 1.11, 5.84 ± 3.44, 2.47 ± 0.32, 7.32 ± 3.82, and 2.47 ± 0.85 (*p* > 0.05), while *FGF2*, *FGF7*, and *FGF13* were downregulated by the folds of 0.18 ± 0.06, less than 0.01, and 0.21 ± 0.06 with WNT activation by CHIR99021 (*p* < 0.01; Fig. 2b).

Among them, FGF7 and FGF9 were of particular interest as their roles in HF formation/maintenance have been examined previously [5, 6]. Intriguingly, *FGF7* and *FGF9* were dose-dependently down- or upregulated by the addition of CHIR99021 (Fig. 2c). Of note, downregulation of *FGF7* was remarkable, presenting more than 100-fold difference between WNT-activated and non-activated sFBs. This differential expression was confirmed at protein level by western blot (Fig. 2d). Considering the suppressive role of FGF7 in HF formation [12] and the hair inductive effect of FGF9, the findings supported the idea that sFBs change their FGF expression profiles to be folliculogenic upon WNT activation.

### 3.3. Expression of representative HF biomarkers in singly or co-cultured KCs and DPCs with FGF7 or FGF9

**Fig. 3:**
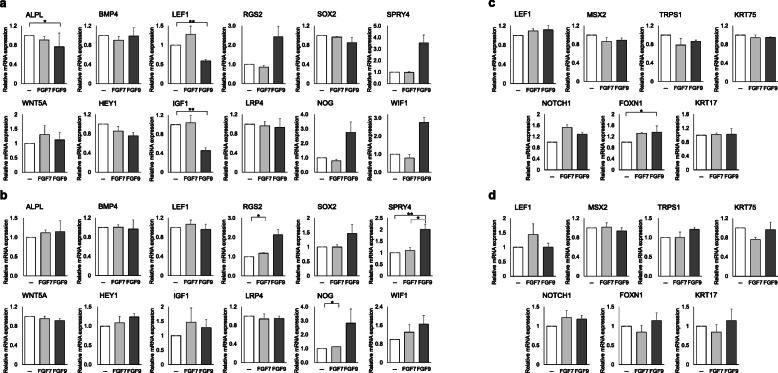
Expression of biomarker genes in human dermal papilla cells and keratinocytes with or without FGF7 and FGF9. Expression of dermal papilla cell (DPC) biomarkers in DPCs in DPC single culture (**a**) and keratinocyte (KC) and DPC co-culture (**b**). Expression of hair follicle (HF) biomarkers in KCs in KC single culture (**c**) and KC-DPC co-culture (**d**). *p < 0.05. **p < 0.01. The data were obtained from four experiments for **a**, **b**, and **d** and three experiments for **c**

To further examine the influence of FGF7 and FGF9 on HFs, which were differentially expressed in sFBs upon WNT activation, DPCs and KCs were singly and subsequently co-cultured in the medium supplemented with respective FGFs, and the expression of representative biomarkers of each cell lineage were assessed (Fig. 3a–d). When DPCs were stimulated with FGF9 in single culture, increase in some representative DPC biomarkers, including *RGS2* (2.44 ± 0.54-fold), *SPRY4* (3.52 ± 0.5-fold), and *NOG* (2.75 ± 0.74-fold) were observed (Fig. 3a) (*p* > 0.05). This tendency was sustained when DPCs were co-culture with KCs (Fig. 3b). Interestingly, DPCs co-cultured with KCs restored well-established DPC markers, *ALPL*, *LEF1*, and *IGF1* (Fig. 3b), which were rather suppressed by FGF9 in singly cultured DPCs (Fig. 3a). Influence of FGF7 on DPC biomarker expression in singly cultured DPCs was not remarkable (Fig. 3a), while a subtle increase of two DPC biomarkers, *RGS2* (1.18 ± 0.03-fold) and *NOG* (1.12 ± 0.01-fold), was observed when DPCs were co-cultured with KCs (*p* < 0.05; Fig. 3b). These findings suggested that FGF 7 and 9 may functionally ameliorate human DPCs especially when they are coexisted with KCs.

In contrast, the effect of FGF7 and FGF9 on KC biomarker expression was not evident except for moderate upregulation of *FOXN1* only in FGF9-stimulated singly cultured KCs (1.32 ± 0.32-fold) (Fig. 3c).

### 3.4. Effect of FGF7/9 on hair follicle induction in hair chamber assay

**Fig. 4:**
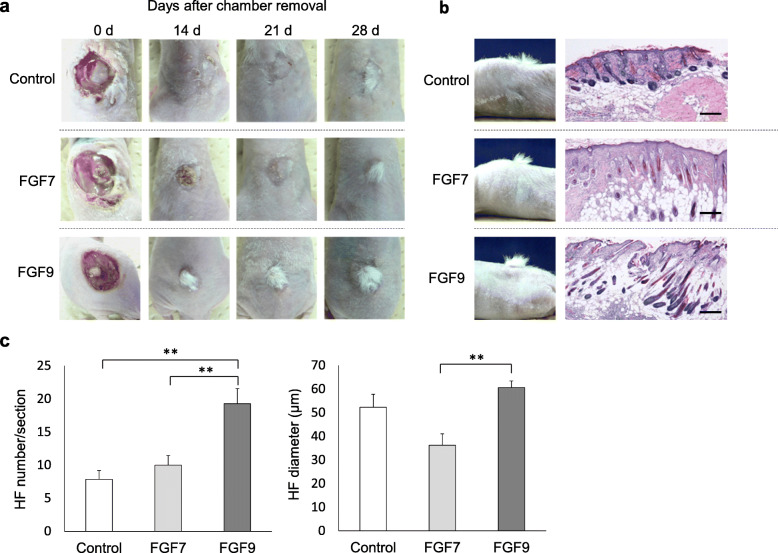
Effects of FGF7/9 on hair induction in vivo hair reconstitution assay. **a** Representative time course of re-epithelization and hair regeneration after the removal of chamber in control and FGF7- and FGF9-treated groups were presented. **b** Representative histopathology of re-epithelized skin 6 weeks after the removal of chambers in three groups. Terminal hairs were remarkable in the FGF9-treated group, whereas hair follicles observed in the control and the FGF7-treated groups were comparatively immature. Scale bars = 10 μm. **c** Regenerated hair follicle (HF) density (total HF number per one vertical section) and the average HF diameter in three groups. FGF9 group presented increase in both the number and the diameter of newly formed HFs. *p < 0.05. **p < 0.01. The data were obtained from seven respective experiments.

As stable in vivo reconstitution of human HFs remains challenging, mouse chamber hair reconstitution assay was adopted to investigate the effect of FGF7 and FGF9 on HF regeneration. Mice in all groups started to grow visually detectable hairs 8 days at the earliest after the chamber removal but the amount of hair detectable on FGF 9-treated mice became obvious in 2 weeks when compared with control and FGF7-treated mice (Fig. 4a). In histopathology, FGF9-treated groups formed more anagen hairs compared to FGF7-treated or control groups (Fig. 4b). In FGF9-treated mice, the number of regenerated HFs (19.29 ± 2.28 per one vertical section) and HF-diameter (60.58 μm ± 2.84) were the greatest among the three groups at 6 weeks after the grafting (Fig. 4c). Although FGF7-treated mice showed tendency to form more HFs (10.00 ± 1.45 per one vertical section) than control mice (7.86 ± 1.32 per one vertical section) without statistical significance, HF diameter (36.22 μm ± 4.79) was smaller than that of control mice (52.29 μm ± 5.50) (Fig. 4c). Therefore, FGF9 promoted in vivo HF regeneration, while the influence of FGF7 on HF forming efficiency was inconclusive.

## 4. Discussion

Despite growing interest in the roles of dermal cells in HF morphogenesis and homeostasis, most investigations have been conducted on DPCs or DSCs, and little attention has been paid to the third HF-associated dermal cells, i.e., sFBs. The role of dermal fibroblasts to support HF formation has been implicated in HF regeneration studies [43]; however, the mechanism by which they promote folliculogeneis has been insufficiently studied. A report by Gay et al. that dermal fibroblasts secrete FGF9 to support wound-induced HF neogenesis [5] prompted us to investigate if human sFBs similarly contribute to HF induction via FGFs.

Our literature search revealed the current understanding of the role of FGFs in the context of HF biology has been quite limited (Table 1). FGF expression profiling in HF-related dermal cell subsets; DPCs, DSCs, and sFBs first suggested a unique FGF expression pattern in each subgroup. In an examined sample, sFBs were characterized by higher expression of *FGF5* and *FGF18*, which individually induce a catagen [20–23] or a telogen [34–36] in animal models, compared to those in DPCs and DSCs. *FGF13* expression was also relatively strong in sFBs. FGF13 has been shown to be preferentially expressed in the bulge epithelial stem cell area and speculated to be involved in the regulation of cell functions [37]. In addition, reduction of *FGF13* expression results in X-linked congenital generalized hypertrichosis [44]. These findings suggested that sFBs by themselves rather suppress HF induction in homeostasis. In our experiment, *FGF7* was diffusely expressed in all three dermal cells. The role of FGF7 on hair follicle is diverse ranging from blockage of hair follicle induction [6] to prolongation of anagen phase [19]. Considering that the sample was taken from mature HFs most likely in the anagen phase, the result supported that these three mesenchymal cells cooperatively work on the maintenance of hair cycle. Distinctively high expression of *FGF1* and *FGF10* in DPCs as compared to DSCs and sFBs could be intriguing and deserves further investigation as both contribute to HF formation and exhibit protective roles in HF homeostasis [16, 29, 30]. As the dataset presented in Fig. 1 was generated from dermal cell populations derived from a single donor, the result cannot be generalized but should be interpreted as an example implying distinct FGF expression profiles among different lineages of dermal cells. The result could be influenced by the age, sex, and other endocrinological status, including androgen levels. To what extent in vitro FGF expression profiles of dermal cell subsets reflected their *bona fide* FGF gene expression profiles in vivo represents an important question to be addressed; however, it is reasonable to conclude that DPCs, DSCs, and sFBs can be distinguished based on their intrinsic FGF expression patterns.

Several previous studies have suggested pivotal roles of canonical WNT signaling pathways and FGFs in HF regeneration and neogenesis [5]; however, their relationship has not been well investigated. In hair neogenesis, canonical WNT and FGF pathways can interact in various manners. Beta-catenin, a pivotal effector in canonical WNT signaling pathway, directly upregulates some FGF genes, at least including *FGF10*, *18*, and *20*, via the activation of TCF/LEF, transcription factors located in the downstream of β-catenin [45, 46]. Moreover, Some FGFs further accelerate WNT signaling by a positive feedback loop system. For example, Fgf9-Wnt2a feedback loop in dermal fibroblasts has been suggested to enhance wound-induced hair neogenesis in mice model [5]. The upregulation of these FGFs via WNT activation was demonstrated in our experiment, supporting these mechanisms. The relationship between FGFs and WNT signaling can be more complex in a certain tissue environment [47]. In urine-derived renal progenitors, WNT activation upregulates TGF-β pathway, which leads to the downregulation of *FGF2* [48]. If the same machinery underlies hair neogenesis remains elusive; however, the downregulation of *FGF2* via WNT activation in our experiment is consistent with this observation.

Among examined FGFs, FGF7 and FGF9 were of particular interest as their potentially conflicting roles in HF biology have been well documented [5, 6, 19] and further experimentations clearly demonstrated that the changes were dose-dependent. Upregulation of *FGF9* in sFBs responding to WNT activation can be interpreted as an observation analogous to that reported in murine dermal fibroblasts [5] and may be linked to pro-folliculogenic activity in human sFBs. Interpretation of marked decrease in *FGF7* expression in WNT-activated sFBs would not be that straightforward. FGF7 is an established DP biomarker which promotes the proliferation of hair germ/matrix cells and initiates a new hair cycle [24], rather suggesting that FGF7 could support HF morphogenesis. However, FGF7 both time- and dose-dependently inhibits the formation of primary pelage and whisker HF in mouse skin [6]. In the mice embryonic skin, Wnt signaling is prominent during E14.5–15.5, when HF placodes are formed [49]. Thus, robust downregulation of *FGF7* by WNT activation could enhance HF formation.

FGF9 upregulated *RGS2*, *SPRY4*, and *NOG* in DPCs. *RGS2* expression has been reported to correlate with hair inductive capacity of human DPCs [12], yet its role in HF biology has not been well understood. SPRY4 is a known inhibitor of FGF10 and shown to be regulated in association with FGF10 expression, which was increased by CHIR99021 in sFBs. Upregulation of *NOG* is a notable finding as dermal-derived NOG has been shown to induce HFs in murine model of HF morphogenesis [50]. Unexpectedly, *ALPL*, *LEF1*, and *IGF1*, representative markers reflecting functional activities of human DPCs, were downregulated by FGF9; however, their expression levels were restored to levels comparable to controls when co-cultured with KCs. These findings might be attributed to intrinsic biological properties of DPs. In DPC single culture, several biomarker genes were shown to be downregulated, which could be restored after WNT activation [12]. In the current experimental setting, FGF9 might have downregulated *ALPL* or *LEF1* via negative feedback as a consequence of putatively potent WNT activation specific to DPCs. DPCs may be able to enjoy optimal WNT-mediated folliculogenic effect of FGF9 only under the co-existence of KCs, mimicking in vivo microenvironment. In contrast, the effects of FGF7 on DPCs alone or DPC-KC co-culture were moderate, suggesting that the influence of drastic WNT-induced *FGF7* downregulaion on HF formation would be minimal.

In line with the observations obtained in co-culture experiments, FGF9 increased HF forming efficiency in the chamber assay, while such promotive effect was minimal in FGF7-treated transplants. The average diameter of newly formed HFs was larger in FGF9-treated group compared to control or FGF7-treated group. This data needs to be carefully interpreted as the increase in diameter can result from change in hair cycle, namely, accelerated or prolonged anagen phase, and does not necessarily mean the formation of larger HFs. Still, the findings favorably support the functionality of FGF9 to promote HF formation.

We are aware of the limitations of this study. The effect of in vitro expansion on FGF expression profiles of individual dermal cell subsets was insufficiently assessed. DPCs and DSCs have been considered to be heterogenous, which represents important future topic to be investigated. Ideally, human scalp samples can be snap frozen on the site and sectioned for total RNA collection via laser capture microdissection from individual dermal cells [51]. However, this approach is technically very challenging to collect sufficient amount of high-quality RNA from scattering sFBs in vivo. The magnitude of WNT activation in HF morphogenesis would be different from CHIR99021 stimulation adopted in this study. The use of recombinant WNTs, represented by WNT3A, in parallel with CHIR99021 would be beneficial to assess the potency required for eliciting trichogenic activities. FGF7 or FGF9 concentrations in co-culture experimentation would not be equivalent to those secreted from WNT-activated sFBs in vivo. Finally, mouse cells are exclusively used in the chamber assay which indirectly supported the scenario that FGF7 and FGF9 from dermal fibroblasts promotes HF formation. Recent studies reported that the combination of neonatal human KCs with carefully inspected trichogenic human DPCs successfully yielded human HF structures in the chamber assay [52, 53]. Perhaps, sFBs can be mixed with similar HF inductive human cells and used for the chamber assay. Once such approach is established, inhibition of WNT signaling, FGF7, or FGF9 in sFBs would allow more direct demonstration of the supportive effect of sFBs in HF formation.

## 5. Conclusion

The findings in this study demonstrated previously less recognized differential FGF expression profiles in HF-related dermal cell subsets including sFBs, and unreported robust FGF expression change upon WNT activation in sFBs. Taken together, the current observations support the concept that enhancement of WNT-FGF9 axis in perifollicular fibroblasts which may provide a strategy to achieve successful human HF regeneration.

## Supplementary information

**Additional file 1. Supplementary Table 1.** Sequence of primers used for RT-PCR. **Supplementary Table 2.** Sequence of primers used for quantitative RT-PCR are included.

## Data Availability

The datasets used and/or analyzed during the current study are available from the corresponding author on reasonable request.

## Abbreviations

ALPL: Alkaline phosphatase liver/bone/kidney
BMP4: Bone morphogenetic protein 4
DMSO: Dimethyl sulfoxide
DPC: Dermal papillae cell
DSC: Dermal sheath cell
FB: Fibroblast
FBS: Fetal bovine serum
FOXN1: Forkhead box N1
GAPDH: Glyceraldehyde-3-phosphate dehydrogenase
HEY1: Hairly/enhancer-of-split related with YRPW motif 1
HF: Hair follicle
HG: Hair germ
IGF1: Insulin-like growth factor 1
KC: Keratinocyte
KRT17: Keratin 17
KRT75: Keratin 75
LEF1: Lymphoid enhancer-binding factor
LRP4: LDL receptor-related protein 4
MSX2: msh homeobox 2
NOG: noggin
ORS: Outer root sheath
PBS: Phosphate-buffered saline
PCR: Polymerase chain reaction
RGS: Regulator of G protein signaling 1
RT: Reverse transcription
SDS: Sodium dodecyl sulfate
sFB: Scalp-derived fibroblast
SOX2: SRY-box 2
SPRY4: Sprouty RTK signaling antagonist 4
TRPS1: Transcriptional repressor GATA binding 1
WIF1: Wnt inhibitory factor 1
WNT5A: Wnt family member 5A
