# Supplementary material for: Altered FGF expression profile in human scalp-derived fibroblasts upon WNT activation: implication of their role to provide folliculogenetic microenvironment
Source: Inflamm Regen. 2020 Sep 21;40:35. doi: 10.1186/s41232-020-00141-8 (PMC7507293; doi:10.1186/s41232-020-00141-8)
Supplement: Supplementary file 1 — Additional file 1. Supplementary Table 1. Sequence of primers used for RT-PCR. Supplementary Table 2. Sequence of primers used for quantitative RT-PCR are included. [file 41232_2020_141_MOESM1_ESM.docx]

**Supplementary Table 1 Sequence of primers used for RT-PCR**

| **Gene** | **Forward primer (5' to 3')** | **Reverse primer (5' to 3')** |
| --- | --- | --- |
| **β-actin** | TCGTGCGTGACATTAAGGAG | TTGCCAATGGTGATGACCTG |
| **FGF1** | TGTGGAGAGAGGTACAGCCC | AAGGTGGTGATTTCCCCTTC |
| **FGF2** | GGAGAAGAGCGACCCTCAC | AGCCAGGTAACGGTTAGCAC |
| **FGF5** | CGAGGAGTTTTCAGCAACAA | TTGAAAACGCTCCCTGAACT |
| **FGF7** | GGGACCCAAGAGATGAAGAA | TTCACTTTCCACCCCTTTGA |
| **FGF10** | AGAAGAACGGGAAGGTCAGC | TGCTGTTAATGGCTTTGACG |
| **FGF18** | ACTTCCTGCTGCTGTGCTTC | CTTACGGCTCACATCGTCC |
| **FGF9** | TATTTCGGTGTGCAGGATGC | TTAACAAAACCGGGCTGTCC |
| **FGF16** | AACGTGCCCTTAGCTGACTC | CTTCAGGTGGGCGAAGTCT |
| **FGF20** | CAGCTCTATTGCCGCACC | AGTCCCACTGCCACACTGAT |
| **FGF13** | GTCTGCGAGTGGTGGCTATC | TGAATTTGCACTCAGGTGTGA |

**Supplementary Table 2 Sequence of primers used for quantitative RT-PCR**

| **Gene** | **Forward primer (5' to 3')** | **Reverse primer (5' to 3')** |
| --- | --- | --- |
| **β-actin** | TCGTGCGTGACATTAAGGAG | TTGCCAATGGTGACCTG |
| **FGF1** | TGTGGAGAGAGGTACAGCCC | AAGGTGGTGATTTCCCCTTC |
| **FGF2** | GGAGAAGAGCGACCCTCAC | AGCCAGGTAACGGTTAGCAC |
| **FGF5** | CGAGGAGTTTTCAGCAACAA | TTGAAAACGCTCCCTGAACT |
| **FGF7** | GGGACCCAAGAGATGAAGAA | TTCACTTTCCACCCCTTTGA |
| **FGF10** | AGAAGAACGGGAAGGTCAGC | TGCTGTTAATGGCTTTGACG |
| **FGF18** | ACTTCCTGCTGCTGTGCTTC | CTTACGGCTCACATCGTCC |
| **FGF9** | TATTTCGGTGTGCAGGATGC | TTAACAAAACCGGGCTGTCC |
| **FGF16** | AACGTGCCCTTAGCTGACTC | CTTCAGGTGGGCGAAGTCT |
| **FGF20** | CAGCTCTATTGCCGCACC | AGTCCCACTGCCACACTGAT |
| **FGF13** | GTCTGCGAGTGGTGGCTATC | TGAATTTGCACTCAGGTGTGA |
| **ALPL** | ATTGACCACGGGCACCAT | CTCCACCGCCTCATGCA |
| **BMP4** | GCCCGCAGCCTAGCAA | CGGTAAAGATCCCGCATGTAG |
| **LEF1** | CCCGATGACGGAAAGCAT | TCGAGTAGGAGGGTCCCTTGT |
| **RGS2** | GACTGCAGACCCATGGACAA | TCGCTTCTCCTCGCTCTTG |
| **SOX2** | TGCGAGCGCTGCACAT | TTCTTCATGAGCGTCTTGGTTTT |
| **SPRY4** | TTGGTGCAGGGCATCTTCTAC | GCGCAGGAGCCCTCATC |
| **WNT5A** | TCCACCTTCCTCTTCACACTGA | CGTGGCCAGCATCACATC |
| **HEY1** | GCGCACGCCCTTGCT | GCCAGGCATTCCCGAAA |
| **IGF1** | AAGGAGGCTGGAGATGTATTGC | CGGACAGAGCGAGCTGACTT |
| **LRP4** | GGCACAGCCACTAGGTTTTAACA | GAAGGCCGAGGCAAGCA |
| **NOG** | CTGGTGGACCTCATCGAACA | CGTCTCGTTCAGATCCTTTTCCT |
| **WIF1** | TGGCATGGAAGACACTGCAA | GGCCTCAGGGCATGTATGA |
| **MSX2** | GGGCCAAGGCGAAAAGA | GCAGCCATTTTCAGCTTTTCC |
| **TRPS1** | TGAATCCCAGTCCCTGTTACG | GGCAATTGGCACAAAAAACAC |
| **KRT75** | AGGACTGTGAGGCAGAACCTAGA | CCGTCGGAGCTCACTGGTA |
| **NOTCH1** | GGTGAGACCTGCCTGAATG | GTTGGGGTCCTGGCATC |
| **FOXN1** | CGGAGCACTTTCCTTACTTCA | CTCCACCTTCTCGAAGCACT |
| **KRT17** | AATTGAGGAGCTGCAGAACAA | AAACTTGGTGCGGAAGTCAT |
